# Supplementary material for: Mechanism of DNA capture by the MukBEF SMC complex and its inhibition by a viral DNA mimic
Source: Cell. Author manuscript; Available in PMC 2025 Jul 4. (PMC7617805; doi:10.1016/j.cell.2025.02.032)
Supplement: Supplementary Figures [file EMS206515-supplement-Supplementary_Figures.pdf]

CellPress
OPEN ACCESS

Supplemental figures

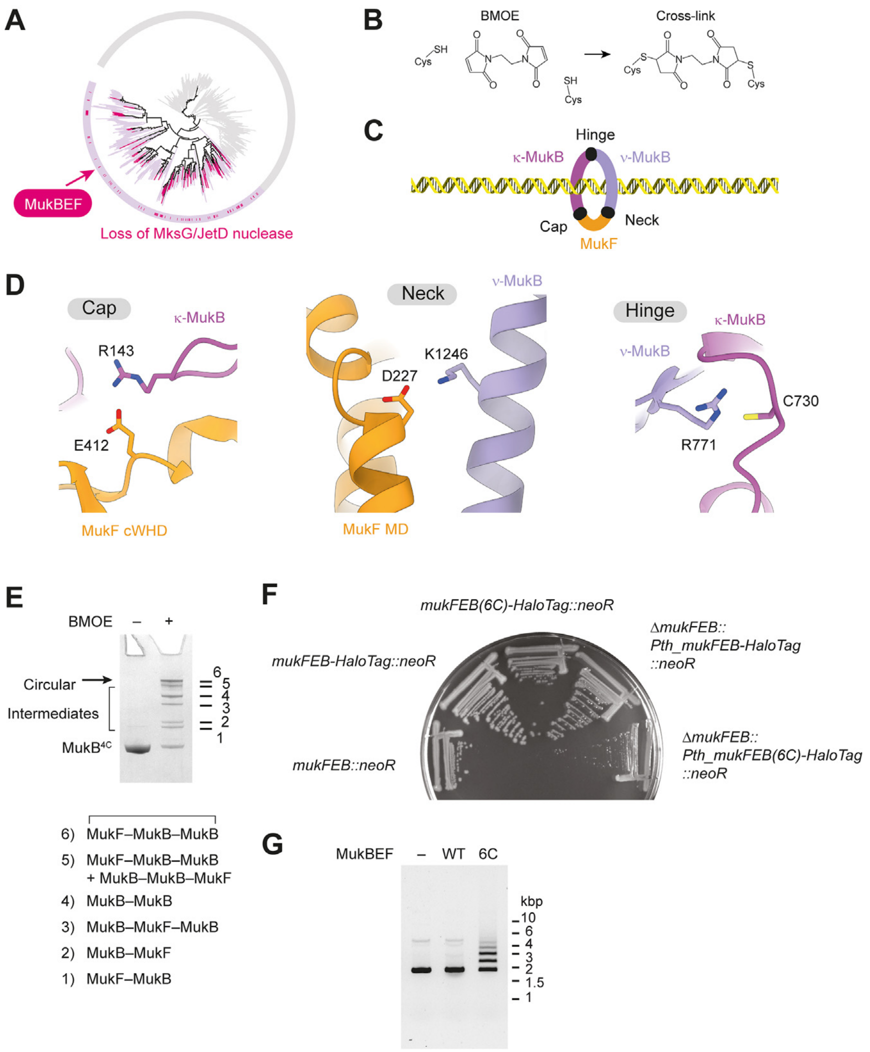

Figure S1. Phylogeny of MukBEF and cysteine mutagenesis, related to Figure 1

(A) Loss of the MksG/JetD nuclease across the Wadjet group. Absence of the nuclease gene is shown on the phylogenetic tree from Figure 1A.

(B) BMOE crosslinking reaction between cysteine pairs. A covalent bridge between the cysteine sulfur atoms is formed.

(C) Location of the three potential gates shown in the simplified cartoon representation of MukBEF.

(D) Location of the residues in *P. thracensis* MukBEF targeted by cysteine mutagenesis. Residues are shown in the apo state (PDB: 7NYY).

(E) Product assignment of the cross-linking reaction shown in Figure 1D, inferred from the closely related band pattern observed for *E. coli* MukBEF^{6C} *in vivo*.[18]

(F) Growth of *E. coli* strains with the *P. thracensis mukFEB* locus substituted for the endogenous *mukFEB* locus. Strains were streaked for single colonies on TYE and grown for 14 h at 37°C. Note that the cysteine mutant *P. thracensis* variant causes a mild growth defect. Strains used: SFB012, SFB017, SFB174, SFB208, and SFB209.

(G) DNA loading assay with *P. thracensis* MukBEF and MukBEF^{6C}. Reactions were performed with 5 mM ATP as in Figure 1F. Formation of the DNA ladder requires the presence of the engineered cysteines.

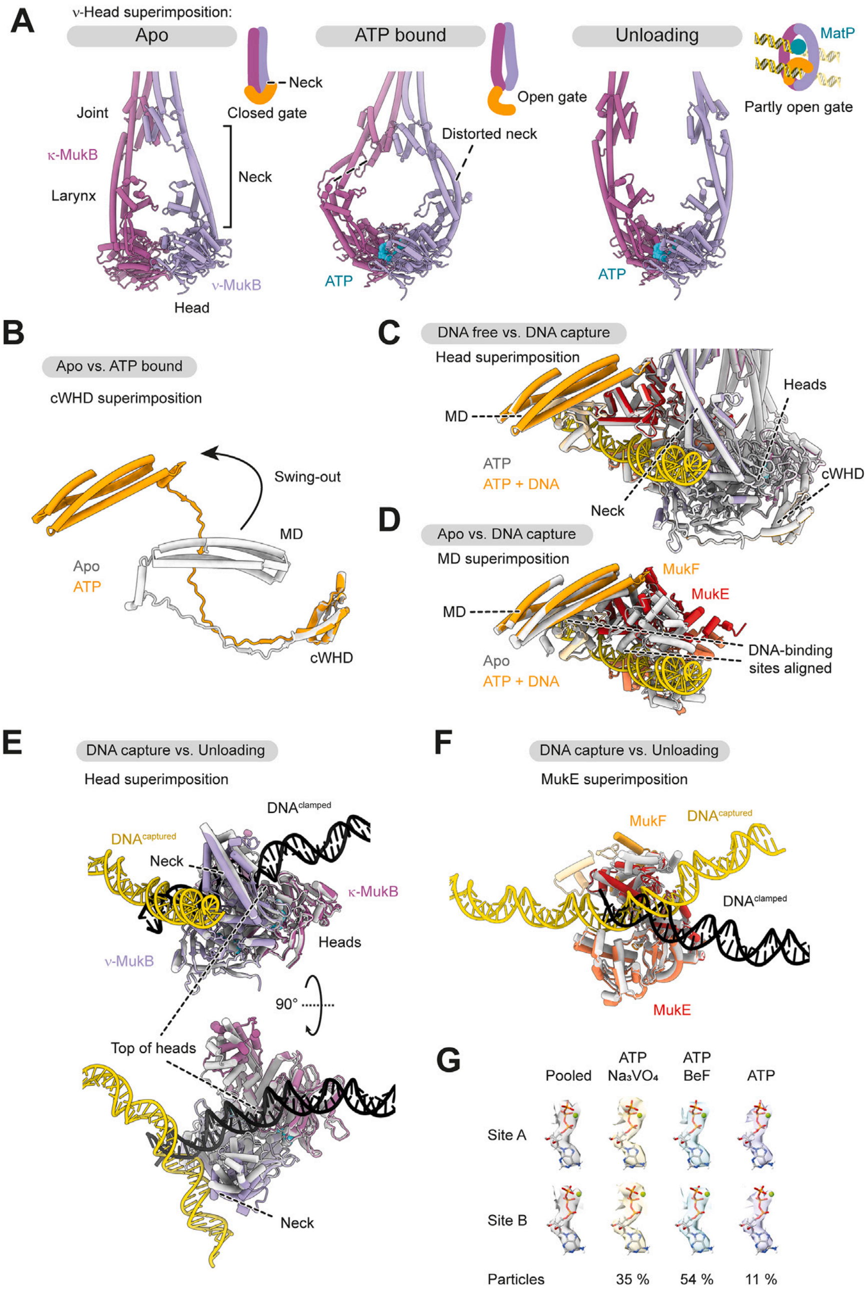

Figure S2. MukBEF conformations and DNA binding, related to Figure 2

(A) Conformations of the MukB head and neck region in the apo state (left; PDB: 7NYY), ATP-bound open-gate state (middle; PDB: 9GM6), and DNA-bound unloading state (right; PDB: 7NYW). The open-gate state has a severely distorted neck.

(B) Comparison of MukF in apo state (gray; PDB: 7NYY) and ATP-bound open-gate state (colored; PDB: 9GM8). The MD swings out upon ATP binding. Structures were superimposed on the cWHD.

(C) Comparison of the open-gate state (gray; PDB: 9GM8) and capture state (colored; PDB: 9GM9). Structures were superimposed on the heads.

(D) Comparison of the apo state (gray; PDB: 7NYY) and capture state (colored; PDB: 9GM9). Structures were superimposed on the MD. The DNA-binding surfaces of MukE and MukF align in the capture state.

(E) Comparison of DNA capture state (colored; PDB: 9GM8) and DNA unloading state (gray, black; PDB: 7NYW). Structures were superimposed on the heads.

(F) Comparison of DNA binding to MukE in the DNA capture state (colored; PDB: 9GM9) and DNA unloading state (gray, black; PDB: 7NYW). Structures were superimposed on the MukE dimer.

(G) Comparison of nucleotide cryo-EM density for reconstructions from individual datasets. The structure was refined against the pooled dataset, and individual maps were reconstructed using the particle poses obtained from this consensus refinement. Density in a zone of 2.5 Å around the nucleotide of both ATPase sites is shown, and the fraction of particles in the respective dataset is indicated. The nucleotide was modeled as MgATP.

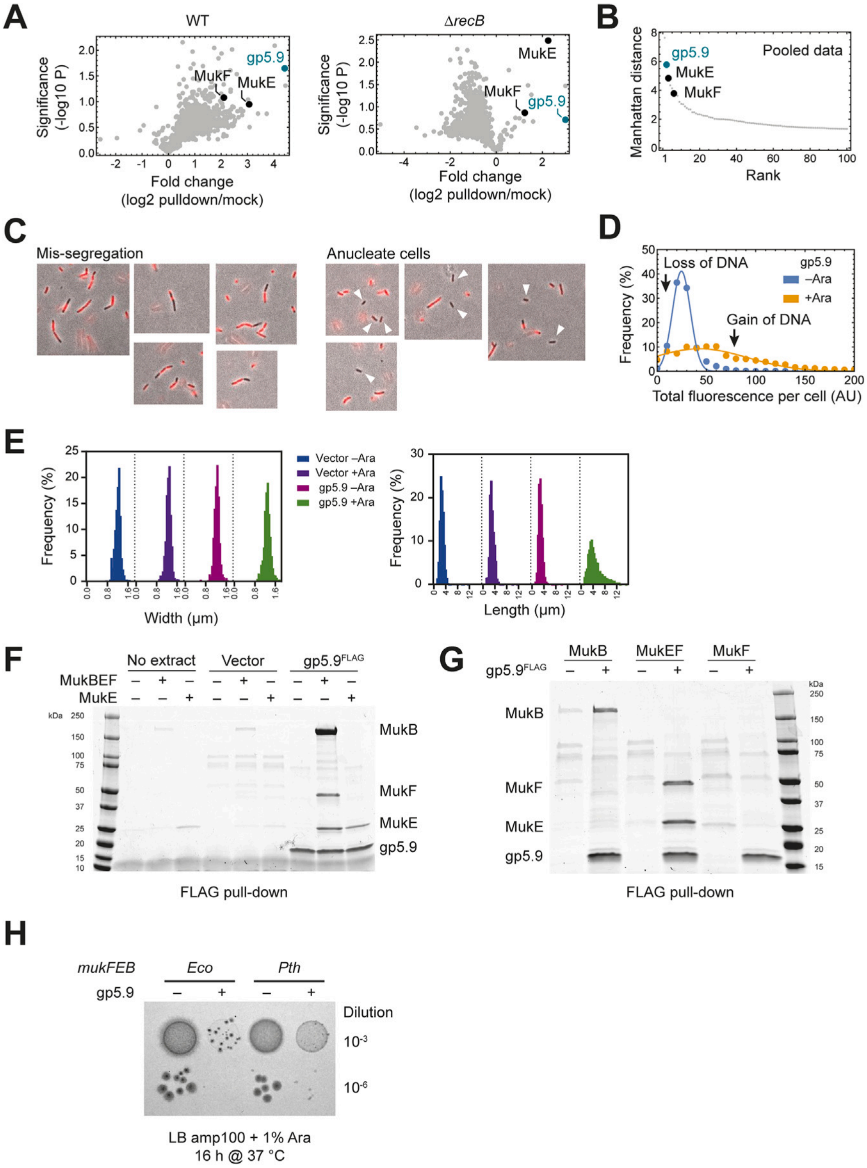

Figure S3. Effects of gp5.9 expression in *E. coli*, related to Figure 3 and Data S2

(A) TMT-MS analysis as in Figure 3C, showing unpooled data for WT and Δ*recB* extracts.

(B) Manhattan distance ranking of the data points shown in Figure 3C.

(C) Examples of chromosome mis-segregation and anucleate cell formation in cells expressing gp5.9. Anucleate cells are indicated by white triangles.

(D) DAPI intensity distributions as in Figure 3E, comparing uninduced and induced conditions.

(E) Cell width (left) and length (right) distributions of the experiment shown in Figure 3D.

(F) Full gel shown in Figure 3F, also showing a pull-down of recombinant protein in the absence of extract.

(G) Pull-down as in Figure 3F, using MukB, MukEF, and MukF proteins.

(H) gp5.9 sensitivity of *E. coli* with the endogenous *mukFEB* locus (*Eco*) replaced by the *P. thracensis* locus (*Pth*). Strains contained an ampicillin-selectable empty vector control or produced gp5.9 from an equivalent arabinose inducible construct. The indicated dilutions were spotted on LB media plus ampicillin with arabinose and incubated at 37°C. While the *Eco* strain only produced few colonies at the low dilution, the *Pth* strain produced a lawn at the same dilution, and single colonies at the low dilution. Note that the *Pth* strain still showed a growth phenotype upon gp5.9 induction, likely due to residual inhibition of MukBEF or inactivation of other targets such as RecBCD. Strains used: SFB289, SFB290, SFB292, and SFB293.

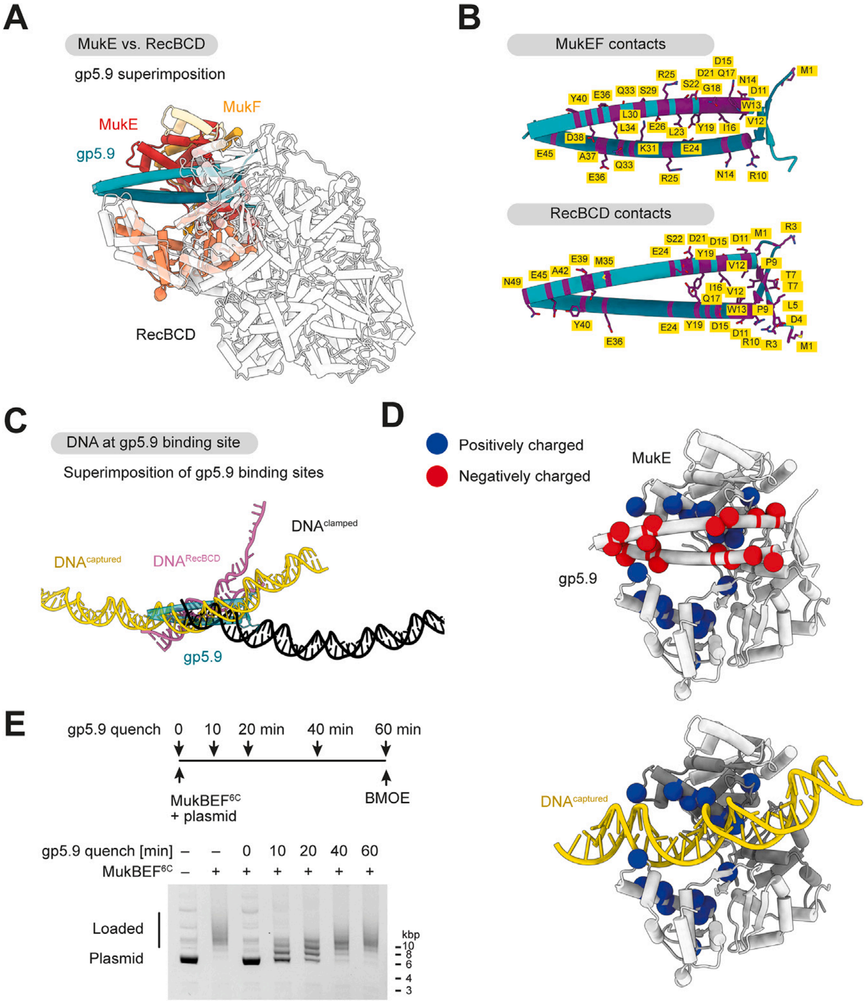

Figure S4. gp5.9 binding of RecBCD and MukBEF, related to Figure 4

(A) Comparison of gp5.9 binding to MukEF (PDB: 9GMD) and RecBCD (PDB: 8B1R).[43] Structures were superimposed on gp5.9.

(B) Binding residues on gp5.9 in the MukEF structure (top; PDB: 9GMD) and the RecBCD bound form (bottom, PDB: 8B1R). Residues with an inter-model atom-atom center distance of less or equal than 4 Å are highlighted in purple.

(C) Comparison of DNA paths at the gp5.9-binding site. gp5.9-bound MukEF and RecBCD were superimposed on gp5.9 as in (A), and then DNA-bound forms were superimposed onto MukE or RecB, respectively. DNA paths are shown for the MukBEF DNA capture state (yellow; PDB: 9GM9), DNA-bound RecBCD (pink; PDB: 5LD2),[97] and the MukBEF DNA unloading state (black; PDB: 7NYW).[18] Superimposed gp5.9 (teal) are shown for reference.

(D) gp5.9 places negatively charged residues (red) close to positively charged residues (blue) in the MukE DNA-binding cleft. C-alpha positions are shown as colored spheres (gp5.9: D11, D15, D21, E24, E36, D38, E43, and E45; MukE: R140, K150, K154, R156, R161, R163, R164, and R179; MukF: R322). The position of DNA in the capture state is shown for reference (bottom; structures superimposed on MukE).

(E) Quenching of DNA loading by gp5.9. Entrapment assay as in Figure 4C, but an 8-fold molar excess of gp5.9 was added at the indicated timepoints. All samples were BMOE treated 60 min after reaction start.

CellPress
OPEN ACCESS

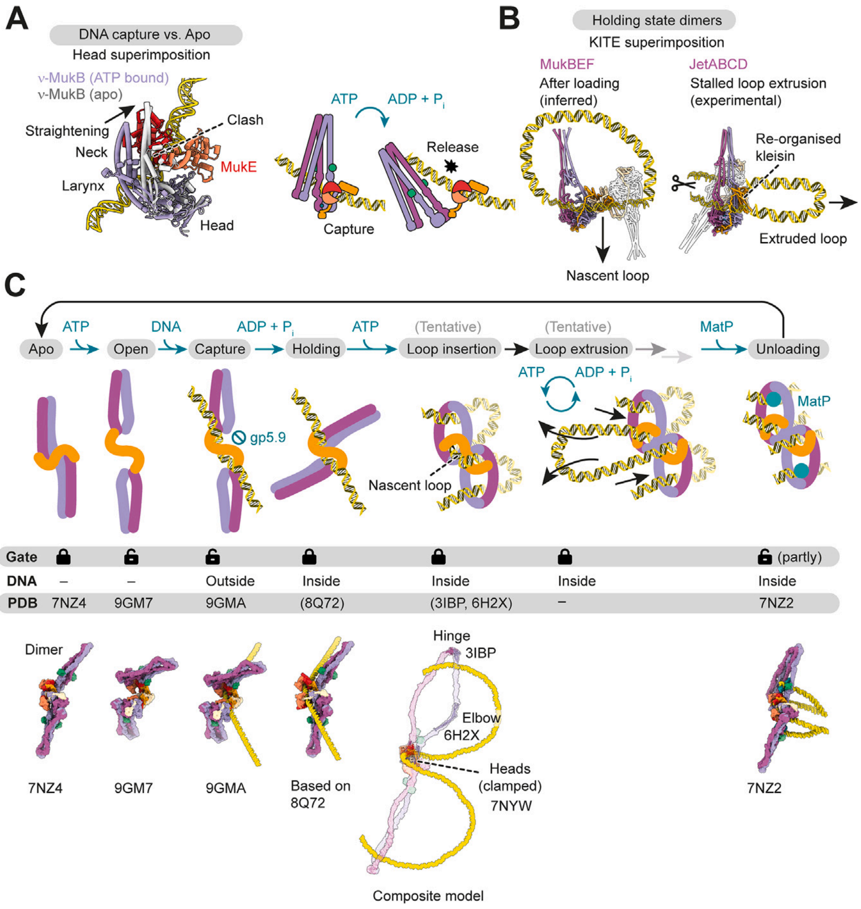

Figure S5. Structural support for DNA loading and loop extrusion, related to Figure 5 and Data S3

(A) Release of MukB upon ATP hydrolysis. A comparison of the DNA capture state (colored) with MukB in the apo state (gray) is shown on the left. Structures were superimposed on the head. Neck straightening in the apo state is incompatible with binding of MukE. This results in release of MukB from DNA-bound MukEF upon ATP hydrolysis, as illustrated on the right. MukB remains attached via the cWHD and linker of MukF.

(B) Comparison of the inferred post-loading holding state of MukBEF and the post-extrusion holding state of *E. coli* Wadjet I (PDB: 8Q72). Models were superimposed on the KITE subunits MukE/JetB of the colored monomer. The second monomer is shown in transparent gray. The captured DNA in the post-loading state corresponds to the extruded loop in the post-extrusion state.

(C) Model of the MukBEF activity cycle as in Figure 5E. Experimental structures and tentative models are shown in the bottom row. The tentative loop insertion state is shown in transparent colors, with experimental sub-structures highlighted in full color. Three-dimensional models for the tentative states are available in Data S3.
